# Challenges and solutions for introducing artificial intelligence (AI) in daily clinical workflow

**DOI:** 10.1007/s00330-020-07148-2

**Published:** 2020-08-14

**Authors:** Elmar Kotter, Erik Ranschaert

**Affiliations:** 1 Department of Radiology, Medical Center – University of Freiburg, Faculty of Medicine, University of Freiburg, Hugstetterstr. 55, D-79106 Freiburg, Germany; 2 Department of Radiology, Elisabeth-Tweesteden Hospital (ETZ), Tilburg, The Netherlands

*“The greatest opportunity offered by AI is not reducing errors or workloads, or even curing cancer: it is the opportunity to restore the precious and time-honored connection and trust”*

*Eric Topol, Deep Medicine: How Artificial Intelligence Can Make Healthcare Human Again*

Artificial Intelligence (AI) is ubiquitous today, and radiology is at the forefront of AI applications in medicine. When participating in a radiology conference, AI is everywhere. Last year’s RSNA AI Showcase hosted more than 120 companies, almost doubling the number of the previous year. In 2019 the Artificial Intelligence Exhibition (AIX) made its grand debut at the ECR, bringing AI to the heart of the technical exhibition. At the scientific meeting a record number of 44 scientific sessions (317 presentations) focused on AI. The number of AI-related abstract submissions both to Radiology journals and to radiological conferences is skyrocketing, reaching 25 % of submissions for Radiology in 2019. It is obvious that there is a hype about AI in radiology.

Many recent publications have shown that AI tools, and especially deep learning (DL), can recognize patterns in medical image data with excellent accuracy. However, there exist some major bottlenecks for the introduction of DL algorithms for diagnostic and routine clinical purposes:

1. As long as supervised learning is used, the availability of high quality annotated datasets required for training is a major hurdle [1, 2].
2. Technical validation: once the algorithm has been trained, it is difficult to prove its robustness and reliability as it works like a black box [3].
3. Another reason why it is difficult to integrate AI algorithms into the radiology workflow is the lack of standards for data sharing between digital systems [3].

Practically, it is very difficult for radiology departments to negotiate contracts for a variety of AI systems and to integrate them all into their IT environment. It becomes even more difficult when hospitals want to use their own algorithms and integrate them seamlessly into their diagnostic workflow. In this way, the situation is reminiscent of the early nineties, when PACS implementations were hampered by the lack of communication standards and information sharing frameworks such as DICOM and IHE. Once these were recognised and applied by most vendors, PACS significantly modified the clinical routine of radiologists and clinicians. Currently however, both the DICOM Standard Committee and the IHE are working on the development of new standards in the domain of AI for radiological workflow.

There are many applications for which most radiologists would welcome the support of AI algorithms, especially concerning tasks related to pattern recognition. Good examples of this are the detection and follow-up of lung nodules in CT’s of the chest, or the analysis of total body CT-scans for the detection and comparison of osteolytic lesions of 5 mm or larger in patients with multiple myeloma. These are repetitive, time consuming tasks without much intellectual challenge (and thus satisfaction) for the radiologist. The development of AI algorithms for medical image analysis was initially mostly driven by computer scientists and software developers for research purposes, which did not always result in solutions that were valuable for radiologists, at least not for clinical application. Slowly but surely, however, the awareness is growing that the demand for useful AI tools ideally comes from the user group, namely the radiologists. For this reason it is important for the radiological community to focus on defining appropriate use cases, based upon existing needs, thus allowing the developers to train algorithms with a clearly clinical purpose.

Probably non-interpretative AI applications such as patient scheduling, prediction of patient affluence or improving image quality will be introduced earlier in clinical practice than diagnostic AI algorithms, because there is no real need to prove their robustness and validity, as is the case with diagnostic AI-based tools. Some of these applications are already commercially available in the latest generation of CT-scanners, such as DL-based image quality improvement for low-dose CT-scans.

In numerous non-scientific media, a kind of myth has been created around the the substitutability of radiologists by artificial intelligence. In most publications, the radiologist's skills are compared with a DL-based system trained for narrow-based tasks, such as the detection of malignant lesions in mammography images. Unlike algorithms, radiologists are experts in interpreting images from different modalities for a wide variety of diseases, and they have a much more holistic view on the patient than any trained algorithm. Radiologists are able to integrate information from many different sources, giving them an indispensable role in finding the best therapeutic options for many patients. In our opinion, AI will augment radiologists in this role, and therefore the abbreviation could also be interpreted as “Augmented Intelligence”. Instead of being considered as stand alone actors, AI systems for diagnostic tasks in radiology should thus be considered as a complement to the radiologist. Studies evaluating these systems should concentrate on measuring and monitoring how the use of AI enhances the performance of radiologists [4].

The introduction of AI in medicine also raises many ethical questions. It is important for radiologists to be actively engaged in the development of ethical and regulatory guidelines for the use and approval of AI-tools in Radiology [3]. Special attention must be paid to the possible built-in bias of algorithms, since this could potentially cause unforeseen harm to patients. In order to timely detect such significant system errors, the overall integration of AI systems in radiology will need standardised and regulated monitoring of outcomes. In a recently published multisociety statement on ethics of AI in radiology it was stated that: “Ethical use of AI in radiology should promote well-being, minimize harm, and ensure that the benefits and harms are distributed among stakeholders in a just manner. We believe AI should respect human rights and freedoms, including dignity and privacy. It should be designed for maximum transparency and dependability. Ultimate responsibility and accountability for AI remains with its human designers and operators for the foreseeable future” [5].

We are currently in the transition phase from a mainly morphological to a functional, quantitative and holistic analysis of image data, supported by rapidly evolving advanced technologies. Within a few decades, systems will become available that think at a human level or higher, allowing us to observe new, as yet unknown phenomena. The radiologist's tasks will be more focused on personalized treatment, including highly specialized and more targeted image-guided tumor biopsies and treatments, and more accurate prediction and evaluation of treatments supported by AI-based analysis of a full spectrum of patient data. In any case, the time has now come to adopt a proactive attitude and to re-evaluate our position as radiologists on the long term.

While still many radiologists fear that this new technology threatens their profession, the younger generation seems to be aware that AI is a revolution for the profession that will improve radiology, and opposes the idea that human radiologists will be replaced. They also see “the need for AI to be included in medical training” [6]. Education on AI for Radiologists should cover both technical and ethical aspects of AI. The new generation of radiologists should not necessarily become computer experts, but they should have a basic knowledge of the underlying technique, so that they can work in partnership with engineers and clinical physicists to train and improve algorithms, preferably with their own data. The situation is comparable to radiologists using MRI: a good understanding of the physical principles is needed to interpret MRI examinations, to know their weaknesses and potential pitfalls and optimise MRI protocols. As was stated by Michael Recht et al: “Radiologists will increasingly become data or information managers” [3], so the training of the younger generation should be adjusted for this purpose.

In our opinion, AI will not weaken radiologists but rather help them strengthen their function and give them an indispensable role in personalised medical care, which finally will positively contribute to the wellness of patients. It is essential however that radiologists acquire sufficient understanding of AI to be able to steer its development and its impact on their profession. Radiologists must therefore become actively involved in this ongoing transformation, in all areas.
